# Bromide of Potassium Pushed to Its Full Extent

**Published:** 1869-11

**Authors:** 


Bromide of Potassium Pushed to its Full Extent.—

The following narrative, not by a member of the profession, is given in the *Brit. Med. Journal.* The narrator is the patient:

It was in June, 1867, that I began taking the bromide; the daily dose being then, I think, about twenty grains. It very soon caused the cessation of the "lapses" (*petit mal*); and in order to make sure, and stop the greater evil also, I went on increasing the dose (hardly with Dr. M.'s permission and yet not against his orders) till at length I should think I must have been taking seventy-grains a day, perhaps sometimes eighty. The first symptom of overdoing the thing that I noticed was the profound and yet disturbed sleep into which it seemed to throw me. I always awoke with a mental struggle and effort, not knowing at first where I was, or what had become of me; in fact, as I told Dr. M., I seemed to have gone too far down into the gulf of sleep. Side by side with this, but, of course less noticeable to me, was the enfeebling of mental power.

A little page in my accounts, which I should usually prepare and balance in half an hour, took me two or three evenings' weary work. But the worst thing was the tendency to talk "Mrs. Malaprop" English, substituting one word ending in "tion" for another, in a most provoking and yet ludicrous way. I had once to write some letters reminding people that their subscriptions were due, and I had the misfortune of havmy letters (I think one or two of them) brought back to me by a clerk, who pointed out to me that I had written "contraction," or some such word, instead of "subscription." I can not just now remember any more instances; but this difficulty in getting and keeping the right word (though the right idea was present to my mind) is very vividly, and not without humiliation, present to my recollection. Soon, of course, my wife and partner saw the change in me, and attributed it to the right cause. I went from home, and for a time dropped the medicine. In a week, my host said, "Why you look ten years younger than when you first came." The stoop in my figure, the slow uncertain speech, and other bad symptoms, especially the heaviness in the eyes, were gone, and I felt quite myself again. I am still taking the medicine, but now never exceed forty grains a day, often taking only twenty; and, if I find the slightest touch of the "Mrs. Malaprop" difficulty, I reduce the dose at once.

